# Use of Facebook by Academic Medical Centers in Taiwan During the COVID-19 Pandemic: Observational Study

**DOI:** 10.2196/21501

**Published:** 2020-11-20

**Authors:** Wei-Min Chu, Gow-Jen Shieh, Shi-Liang Wu, Wayne Huey-Herng Sheu

**Affiliations:** 1 Department of Family Medicine Taichung Veterans General Hospital Taichung Taiwan; 2 Department of Occupational Medicine Taichung Veterans General Hospital Taichung Taiwan; 3 Institute of Health Policy and Management National Taiwan University Taipei Taiwan; 4 School of Medicine National Yang-Ming University Taipei Taiwan; 5 School of Medicine Chung Shan Medical University Taichung Taiwan; 6 Department of Top Hospital Administration Taichung Veterans General Hospital Taichung Taiwan; 7 Division of Endocrinology & Metabolism Department of Internal Medicine Taichung Veterans General Hospital Taichung Taiwan; 8 School of Medicine National Defense Medical Center Taichung Taiwan; 9 Institute of Medical Technology College of Life Science National Chung-Hsing University Taichung Taiwan

**Keywords:** COVID-19, social media, Facebook, medical centers, Taiwan, communication, video post, survey, health promotion, engagement

## Abstract

**Background:**

The battle against COVID-19 remains ongoing, and social media has played an important role during the crisis for both communication and health promotion, particularly for health care organizations. Taiwan’s success during the COVID-19 outbreak is well known and the use of social media is one of the key contributing factors to that success.

**Objective:**

This nationwide observational study in Taiwan aimed to explore the use of Facebook by academic medical centers during the COVID-19 pandemic.

**Methods:**

We conducted a nationwide observational study of all Facebook fan page posts culled from the official accounts of all medical centers in Taiwan from December 2019 to April 2020. All Facebook posts were categorized into either COVID-19–related posts or non–COVID-19–related posts. COVID-19–related posts were split into 4 categories: policy of Taiwan’s Center for Disease Control (TCDC), gratitude notes, news and regulations from hospitals, and education. Data from each post was also recorded as follows: date of post, headline, number of “likes,” number of messages left, number of shares, video or non-video post, and date of search.

**Results:**

The Facebook fan pages of 13 academic medical centers, with a total of 1816 posts, were analyzed. From January 2020, the percentage of COVID-19 posts increased rapidly, from 21% (January 2020) to 56.3% (April 2020). The trends of cumulative COVID-19 posts and reported confirmed cases were significantly related (Pearson correlation coefficient=0.93, *P*<.001). Pages from private hospitals had more COVID-19 posts (362 versus 289), as well as more video posts (72 posts, 19.9% versus 36 posts, 12.5%, *P*=.011), when compared to public hospitals. However, Facebook pages from public hospitals had significantly more “likes,” comments, and shares per post (314, 5, 14, respectively, *P*<.001). Additionally, medical centers from different regions displayed different strategies for using video posts on Facebook.

**Conclusions:**

Social media has been a useful tool for communication during the COVID-19 pandemic. This nationwide observational study has helped demonstrate the value of Facebook for academic medical centers in Taiwan, along with its engagement efficacy. We believe that the experience of Taiwan and the knowledge it can share will be helpful to health care organizations worldwide during our global battle against COVID-19.

## Introduction

### Background

COVID-19 originated from Wuhan, China, and has become a worldwide pandemic with more than 4 million cases and more than 200,000 deaths globally as of May 2020 [1-3]. Following Wuhan’s lockdown [4], most countries with severe outbreaks took multiple measures to contain the virus and stop it from spreading. Steps that were taken included limiting both international and domestic flights, enhancing border controls, and emphasizing the importance of wearing masks and comprehensive hand washing. However, without a much-needed vaccine, the pandemic remains ongoing.

Many scientists have compared COVID-19 with the severe acute respiratory syndrome (SARS) outbreak in 2003 [5]. However, the world we now live in is so extremely different from the world we remember during the 2003 SARS epidemic, as social media now plays such a big role in our lives, more so than any other time in history. Social media has been a valuable tool for more than a decade for both health care organizations and health care professionals, and enables them to connect with people at risk, increase the health literacy of the general population, and improve health outcomes [6-8].

### Prior Work

Thus, as the COVID-19 outbreak unfolded, the characteristics and capabilities of social media have become more evident. Social media directs people to trusted sources, counteracts misinformation, enables connectivity and psychological first aid, advances remote learning, and even accelerates research [9]. Moreover, social media helps to publicly reveal any early warning signals once an outbreak starts [10]. Additionally, the data coming from internet research surrounding social media helps medical professionals and scientists predict outbreaks [11].

Among all available social media, Facebook plays a special role. Reportedly, Facebook has more than 180 million users in the United States and Canada, with that number still increasing [12]. Facebook has not only been used as a communication tool for health care issues, but also as a tool for recruiting participants into medical research studies [13]. During the previous Zika outbreak, Facebook proved that it can be a cost-effective platform for engaging more people to help ensure better disease prevention [14].

### Role of Taiwan Against COVID-19

Taiwan is one of the many countries with a large number of Facebook users [15]. Taiwan’s experience in the fight against COVID-19 is unique, as its low number of cases and fatalities are a result of proactive measures, new technology, and big data analytics [16-18]. Taiwan’s Center for Disease Control (TCDC) used social media widely, mainly the LINE app and Facebook, to maintain surveillance and communicate risk to the public [19].

### Research Gap

Although a previous study showed that social media can be an effective tool for engagement [20], it is not yet clear what type of posts grab more public attention. Similarly, what post timing is the most appropriate to have more engagement such as likes, comments, and shares? In addition, previous studies have shown that video posts had a significant association with favorable engagement rates [12], but how were video posts used on social media by health authorities during a pandemic such as COVID-19 and what were the effects? There is still a research gap when it comes to social media, information dissemination, and disease outbreaks.

There has been no study that has focused on how health care organizations have used social media to communicate with the public and increase health literacy. In Taiwan, medical centers are academic medical facilities providing the highest level of medical care as accredited by the Joint Commission of Taiwan (JCT), under the supervision of the Ministry of Health and Welfare of Taiwan (MOHW). Different from the role of TCDC and the MOHW, medical centers not only educate people for health promotion, but also treat people with illnesses directly. During the COVID-19 pandemic, medical centers in Taiwan have the most responsibility to test and receive patients with COVID-19. Such a “dual role” made medical centers uniquely have bidirectional communication with the public during the pandemic [21].

From previous studies, we understood that the use of Facebook is helpful for raising public awareness and sharing information rapidly and has “listening” characteristics during public health emergencies, such as the Zika outbreak [22]. In addition, a study focused on Facebook use during an Ebola outbreak suggested that information behavior and audience engagement was topic-dependent and regular health promotion efforts should continue taking place during a health crisis [20]. However, we still want to know whether this kind of observation persists in a different context. Does regular health promotion continue taking place during health emergencies? Analyzing the use of Facebook during the COVID-19 pandemic gave us the opportunity to understand the role of social media and the response of health care organizations more clearly.

### Goal of This Study

We believe that social media has played an important role during the worldwide COVID-19 crisis. However, no study has focused on how health care organizations have used social media to communicate with the public and increase health literacy. In a previous study, medical centers in Taiwan ran their social media better than regional hospitals and local community hospitals; thus, we conducted this study as a nationwide observational study involving the use of Facebook data from academic medical centers in Taiwan during the COVID-19 outbreak period.

## Methods

### Data Sources

We conducted a nationwide observational study of all Facebook fan page posts from official accounts linked to all medical centers in Taiwan during the period spanning December 2019 to April 2020. For comparison, we also surveyed all Facebook posts by the MOHW and TCDC.

### Study Group Identification and Data Extraction

We first checked the official names of the medical centers in Taiwan on the JCT website. At the end of 2019, there were 19 medical centers in Taiwan. All Facebook fan page posts by the medical centers, MOHW, and TCDC were created by a branding team from Taichung Veterans General Hospital (TCVGH). The hospital team was established in 2016 and is led by the superintendent of the hospital. The authors searched all Facebook fan pages of the medical centers during the study period by using the names of the hospitals or their abbreviations. If the medical center did not have a Facebook fan page account, it was excluded from the study. We also recorded whether a medical center was a public or private hospital, as well as their location in Taiwan.

### Research Variables

All Facebook posts were categorized into either COVID-19– or non–COVID-19–related posts. A modified focus group reviewed all posts and divided them according to their content characteristics into 14 unique themes according to a previously published study protocol [23]. We then classified the 14 themes into 4 groups for analysis: policy of TCDC, gratitude notes, news/regulations of hospitals, and education. The classification was decided by a modified focus group. A senior leader of our team was appointed as “gatekeeper” of our classification and she would review all posts and their categories to make sure that no classification bias happened. Data regarding each post was also recorded as follows: date of post, headline, number of “likes,” number of messages left, number of shares, video or no-video post, and date of search. Additionally, the number of confirmed cases of COVID-19 was recorded after being taken from public data made available by TCDC. From a previous study, Facebook “Likes” can be used as an indicator of hospital quality [24].

We also categorized all medical centers into different regions and public or private sector. Public hospitals and private hospitals have very different resources in Taiwan. The public sector receives relatively fewer resources from the government despite bearing a heavier burden than the private sector. Personnel and purchasing systems in public institutions are also less flexible, making them less competitive than private hospitals in providing medical services. Thus, better use of social media to promote the hospital itself became more important for public hospitals. That is why we separated all medical centers into public or private classifications. As for the regions, health care in Taiwan is managed centrally by the Bureau of National Health Insurance (BNHI). The divisions of the BNHI are according to region (eg, Northern, Central, Southern, and Eastern) because medical resources are different from one region to another due to geographical differences.

### Statistical Analysis

For the purpose of descriptive analysis, we used the chi-square test to compare the categorized variables. The Kolmogorov-Smirnov test was used to test normality. If the data was nonparametric, the Mann-Whitney U test and Kruskal-Wallis test were used to compare the continuous and categorized variables, respectively. Post hoc analysis with the Dunn post hoc test was performed if there was statistical significance. Pearson correlation coefficients were used to analyze trends of the cumulative COVID-19 posts and cumulative confirmed cases. We used the chi-square test to compare the percentage of video posts among all the medical centers, while Bonferroni correction post hoc tests were performed if there was statistical significance. Statistical significance was set at *P*<.05. All data were analyzed using SPSS (Version 22.0, IBM).

## Results

All Facebook posts from 13 academic medical centers during the period between December 1, 2019, and April 30, 2020, were analyzed. Table 1 shows the scale of the surveyed medical centers during the research period and their Facebook fan page status. In mid-2020, there were 19 medical centers in Taiwan, and among them, 6 were public hospitals, while the others were private. Of them, only 4 medical centers had not established a Facebook fan page. There were 2 other medical centers whose Facebook fan pages had not been renewed for years. Thus, we analyzed a total of 13 medical centers and their Facebook fan page posts during the COVID-19 pandemic. Chung Shan Medical University Hospital had the most COVID-19 posts (95 posts, 14.6%) among all medical centers.

**Table 1:** Nationwide medical center information and Facebook fan pages in Taiwan.

Region, ownership, and name of hospital		Hospital scale^a^		Creation date of Facebook fan page	Number of Facebook fan page “Likes”^b^	Number of COVID-19 Facebook posts, n (%)^c^
		Number of beds	Number of employees			
**Northern, Public**						
	National Taiwan University Hospital	2289	6700	July 8, 2010	10,107	25 (3.8)
	Tri-Service General Hospital	1793	3000	June 30, 2013	13,702	65 (10.0)
	Taipei Veterans General Hospital	2803	6655	February 18, 2014	3098	4 (0.6)
**Northern, Private**						
	Chang Gung Memorial Hospital, Linkou	3700	20,000	—^d^	—	0 (0.0)
	Cathay General Hospital	816	3400	—^d^	—	0 (0.0)
	MacKay Memorial Hospital	938	3600	August 4, 2016	3228	5 (0.8)
	Shin Kong Wu Ho-Su Memorial Hospital	833	2300	March 10, 2020	212	57 (8.8)
	Taipei Municipal Wanfang Hospital	726	1696	January 12, 2018	1676	15 (2.3)
	Far Eastern Memorial Hospital	1383	3369	February 10, 2012	2106	68 (10.4)
**Central, Public**						
	Taichung Veterans General Hospital	1527	4000	January 30, 2012	33,343	66 (10.1)
**Central, Private**						
	Chung Shan Medical University Hospital	1003	2300	March 30, 2017	4945	95 (14.6)
	China Medical University Hospital^e^	1722	4000	April 3, 2012	389	0 (0.0)
	Changhua Christian Hospital	1244	5011	—^d^	—	0 (0.0)
**Southern, Public**						
	National Cheng Kung University Hospital	1189	5035	July 9, 2015	11,891	63 (9.7)
	Kaohsiung Veterans General Hospital	1482	3561	September 10, 2013	17,778	66 (10.1)
**Southern, Private**						
	Chi Mei Medical Center	1278	3664	—^d^	—	0 (0.0)
	Kaohsiung Chang Gung Memorial Hospital^f^	2691	6200	February 16, 2011	7398	0 (0.0)
	Kaohsiung Medical University Chung-Ho Memorial Hospital	1720	4078	November 25, 2019	1548	51 (7.8)
**Eastern, Private**						
	Hualien Tzu Chi Hospital	947	1987	August 20, 2015	8577	71 (10.9)

^a^The data was obtained from the official website of the hospital (access date: April 30, 2020).

^b^The data was obtained from the Facebook fan page of the hospital (access date: April 30, 2020).

^c^The collection period of COVID-19 Facebook posts was from December 31, 2019, to April 30, 2020. Percentages were calculated by taking the number of COVID-19 posts by an individual medical center (n) and dividing it by the total number (N) of COVID-19 posts of all national medical centers (multiplied by 100).

^d^Not available (the official Facebook fan pages were not found).

^e^Date of the most recent Facebook fan page post: November 2, 2012.

^f^Date of the most recent Facebook fan page post: November 15, 2013.

**Figure 1:**
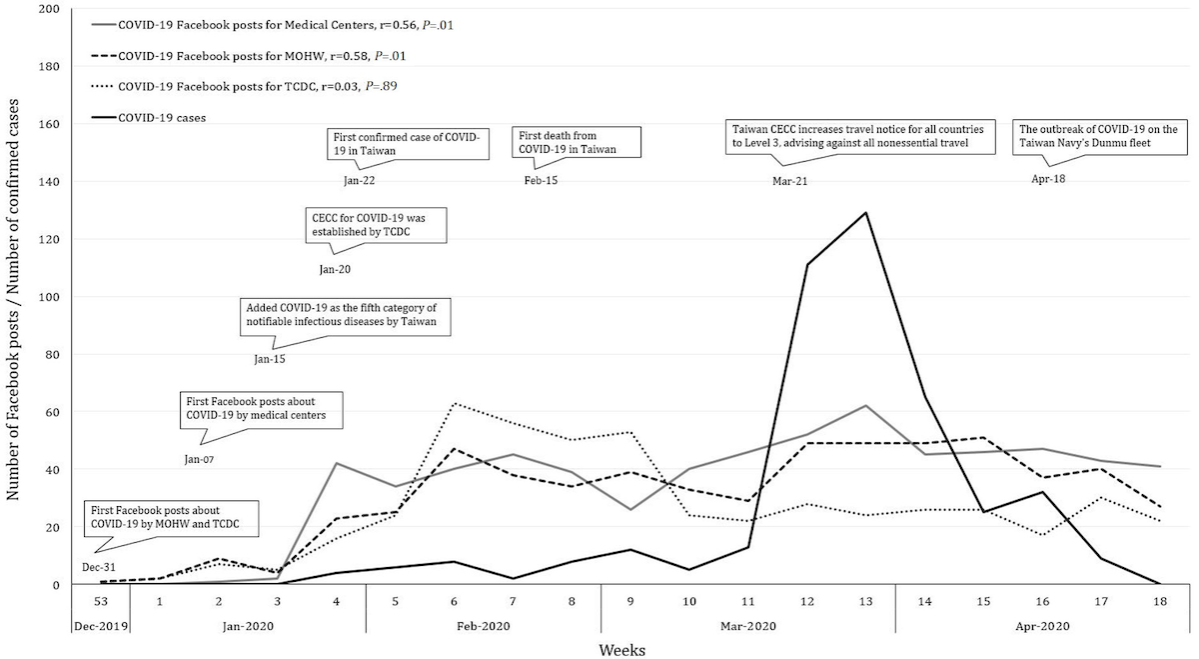
COVID-19 confirmed cases per week in Taiwan and Facebook posts per week by the MOHW, TCDC, and nationwide medical centers from December 2019 to April 2020. CECC: Central Epidemic Command Center; MOHW: Ministry of Health and Welfare; TCDC: Taiwan’s Center for Disease Control.

Figure 1 shows the cumulative COVID-19 posts per week from all academic medical centers, TCDC, and the MOWH, along with the cumulative confirmed cases of COVID-19 per week in Taiwan. There was a relationship between cumulative COVID-19 posts per week from medical centers and confirmed cases per week (Pearson correlation coefficient=0.56, *P*=.01). We also found a similar trend regarding posts from the MOHW (Pearson correlation coefficient=0.58, *P*=.01). However, the relationship between COVID-19 posts by TCDC and confirmed cases was relatively low (Pearson correlation coefficient=0.03, *P*=.89). For most of the study period, medical centers had the most COVID-19–related posts per week; however, posts from TCDC took the lead just before the small outbreak that occurred around March 18, 2020. Multimedia Appendix 1 reveals the cumulative COVID-19 posts from academic medical centers, with the cumulative confirmed cases of COVID-19.

Table 2 shows the trends and distributions of COVID-19–related posts and their categories, along with non–COVID-19 posts. A total of 1816 posts were analyzed during the study period. There were no COVID-19 posts in December 2019 before the outbreak in Wuhan, China. From January 2020, COVID-19 posts increased rapidly, from 21% (January 2020) to 56.3% (April 2020) of total posts. The number of non–COVID-19 posts decreased significantly due to the crowding out effect. Within the 4 categories of COVID-19 posts, at the beginning of the pandemic, most were about National Health Command Center news and regulations (40%); however, as the outbreak continued in February and March, hospital-released news and policy announcements had become the main focus of posts (52% and 36%, respectively). In April, most COVID-19 posts expressed gratitude (42%). After logistic regression surrounding the trend of COVID-19 posts by month, the *P* value for a trend was <.001 (Multimedia Appendix 2). Compared with January, the odds ratio (OR) of COVID-19–related posts of nationwide medical centers was significantly increased in February, March, and April (Multimedia Appendix 3).

**Table 2:** COVID-19 and non–COVID-19 Facebook posts of nationwide medical centers from December 2019 to April 2020.

Type of post		2019	2020				All	*P* value
		December, n (%)	January, n (%)	February, n (%)	March, n (%)	April, n (%)	Total, n (%)	
Total		407 (100.0)	357 (100.0)	308 (100.0)	373 (100.0)	371 (100.0)	1816 (100.0)	
**Disease category**								
	Non–COVID-19 posts	407 (100.0)	282 (79.0)	158 (51.3)	156 (41.8)	162 (43.7)	1165 (64.2)	<.001
	COVID-19 posts	0 (0.0)	75 (21.0)	150 (48.7)	217 (58.2)	209 (56.3)	651 (35.8)	
**Topic category**								
	Policy of Taiwan’s Center for Disease Control	0 (0.0)	30 (40.0)	27 (18.0)	44 (20.3)	23 (11.0)	124 (19.0)	<.001
	Gratitude notes	0 (0.0)	0 (0.0)	5 (3.3)	56 (25.8)	88 (42.1)	149 (22.9)	
	News and regulations from hospitals	0 (0.0)	29 (38.7)	78 (52.0)	80 (36.9)	65 (31.1)	252 (38.7)	
	Education	0 (0.0)	16 (21.3)	40 (26.7)	37 (17.1)	33 (15.8)	126 (19.4 )	

Multimedia Appendix 4 shows the engagement of all COVID-19 posts, including “likes” per post, shares per post, and comments per post. Private hospitals had more COVID-19 posts and more video posts (72 posts, 19.9%) during the research period, but public hospitals had significantly more “likes” (314), comments (5), and shares (14) per post. Regarding different regions, the northern regional medical centers had the most COVID-19 posts (239), while the central regional medical centers had the most “likes” (197) and comments (4) per post. The southern regional hospitals had the most shares per post (14). For video posts regarding COVID-19, the central public medical center had significantly more video posts compared to medical centers of every other region and type.

**Figure 2:**
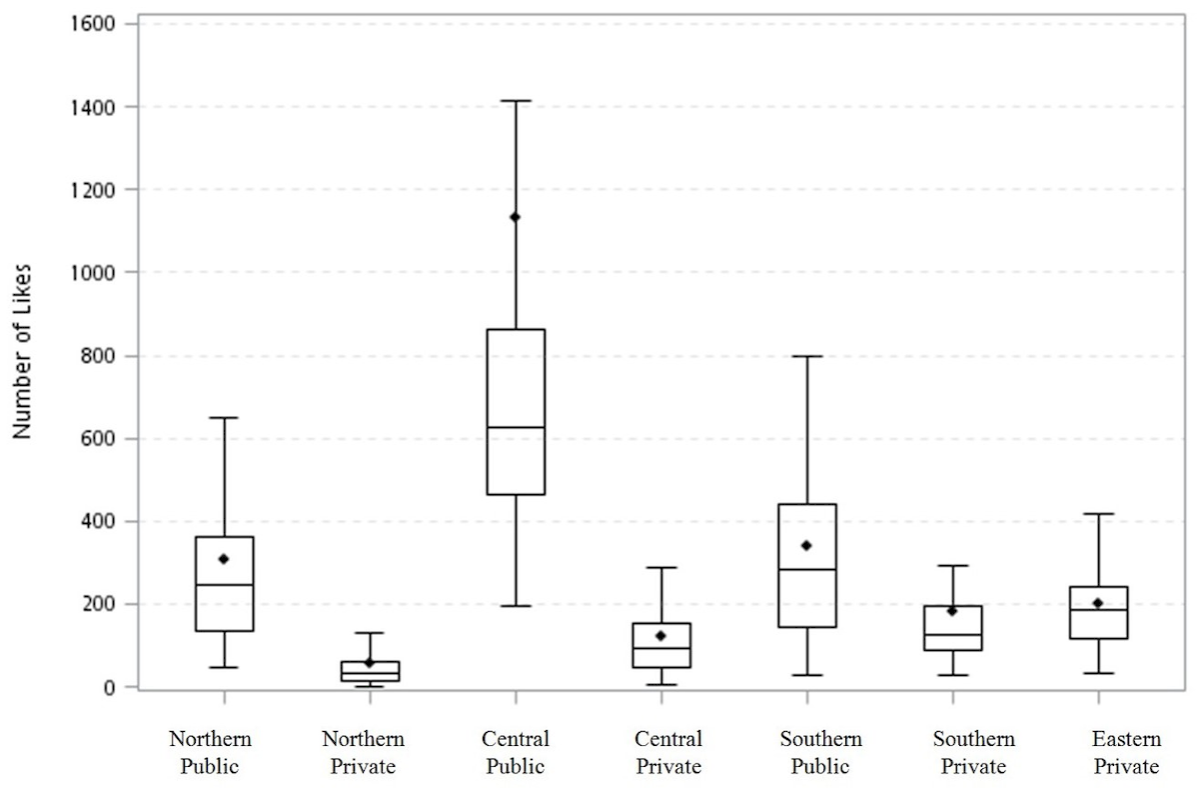
The number of "likes" on COVID-19 Facebook posts by nationwide medical centers in Taiwan, by region and ownership.

Figure 2 shows the trend of “likes” per COVID-19 post for both the different regions and private/public sectors. The central public medical centers had the most “likes” per post, while the northern private hospitals had the fewest “likes” per posts. Multimedia Appendix 5 outlines different post contents and their number of fan likes, comments, and shares, as well as the percentage of video posts published by the central government health agency and 13 nationwide medical centers before and after the COVID-19 outbreak on March 18, 2020. The number of likes and comments increased significantly after the outbreak for the MOHW, TCDC, and medical centers. The number of shares increased significantly after the outbreak only for TCDC and medical centers. We observed that TCDC used more video posts after the outbreak (*P*<.001). Medical centers had more education-related COVID-19 posts compared with TCDC and the MOHW, and those educational posts received more likes and comments after the outbreak. We also analyzed all 4 categories of COVID-19–related posts from each medical center and their effectiveness at achieving engagement (Multimedia Appendix 6).

## Discussion

### Principal Results

To the best of our knowledge, this is the first nationwide study that has explored how academic medical centers in Taiwan used Facebook as a tool for the communication of risk, as well as to increase public health literacy after the outbreak of COVID-19. The main results are as follows:

1. COVID-19 Facebook posts per week increased, along with the scale of public awareness and cumulative cases per week of COVID-19.
2. Non–COVID-19 posts statistically decreased during the COVID-19 pandemic.
3. Public medical centers provided more engagement with the public (through “likes,” comments, and shares), while private medical centers created video posts more often, particularly for the purpose of expressing gratitude.
4. Medical centers in different regions displayed different strategies when using video posts on Facebook.

Although a previous study demonstrated a relationship between social media and infectious disease outbreaks, there was no further analysis of post contents and outcomes. In our study, COVID-19–related posts per week were related to the number of confirmed cases per week, suggesting that medical centers emphasized the disease during the outbreak. However, COVID-19–related posts by TCDC rose before the outbreak of COVID-19, suggesting that TCDC’s response was fast and could be regarded as a “warning” during health crises. Furthermore, gratitude–related and government policy–related COVID-19 posts received many likes and comments, suggesting that these two kinds of posts got more attention. Our findings could be a guide for all medical facilities to have better engagement with the public when public health crises occur.

### Comparison With Prior Work

Social media has been largely used by health care organizations to communicate with the public and improve public awareness surrounding health issues [25], particularly during disease outbreaks such as COVID-19 [26]. A previous study showed that Facebook has been widely used as a tool for both the promotion of health [27,28] and disease management [29,30]. In the United States, most hospitals use their Facebook account as a platform [31]. In addition, a 2017 study revealed that 51.1% (213/417) of hospitals in Taiwan had a Facebook fan page, with an average of 31 posts by the medical centers [24]. Our study offers similar results, with academic medical centers having a higher proportion (13/19, 68%) of Facebook use as a communication tool, and an average post count of 27.9 during the research period.

However, few studies have focused on Facebook use with regard to disease outbreak communication. One study in Malaysia explored Facebook use by health care authorities during the Zika outbreak, showing that health authorities posted updates most frequently within the first two weeks after the outbreak was declared. The findings from our study were different, as we found that the greatest number of COVID-19 posts was published in March 2020, more than one month after the first confirmed case in Taiwan; confirmed cases were increasing rapidly in March. This suggests that public awareness arises from a feeling of imminent threat, and not just from a public declaration made by health care authorities.

As the COVID-19 pandemic is now a worldwide health hazard, health care surge capacity remains an important issue that every country is concerned about [32]. In our study, altered social media content was also noticed, as the number of non–COVID-19 posts rapidly decreased from 100% in December 2019 to 43.7% in April 2020. This discovery implies that much content surrounding health promotion or medical education for chronic diseases such as hypertension or diabetes was not disseminated due to COVID-19 posts capturing the majority of public attention. The long-term effect of such an altered “social media capacity” should be further examined.

We found that medical centers from different regions and with different types of ownership implemented varying strategies when using video posts as a method of communication, with the percentage of video posts pertaining to COVID-19 ranging from 0% to 40.9%. Previous studies found that, when compared with other post types, video posts were the only type that had a significant association with favorable engagement rates, while posts linking shared video posts had a negative impact on engagement [7,33]. Another study revealed that both photos and videos were linked to an increase in the amount of time from the most recent interaction activity [12]. Additionally, video post pairings involving text are easy to remember, and are more likely to capture the attention of the younger generation [34]. Health care organizations should use video posts more often to achieve better engagement and understanding.

Among all regions and types of ownership, one central and public hospital, Taichung Veterans General Hospital (TCVGH), experienced the most “likes” per post (more than 600 on average), and its percentage of video posts was the highest (40.9%).

TCVGH is one of the 19 medical centers in Taiwan and was established in 1982. It is among the medical facilities found in central Taiwan, and is the only government medical center providing medical services to the public. The branding team of VGHTC was first set up in 2016 and they started promoting the hospital branding in 2017; as of May 2020, TCVGH had the largest number of Facebook fans among all medical centers in Taiwan [23]. Its main mission was to establish branding strategies and integrate segmental branding messages. Under the leadership of the hospital’s superintendent, 11 divisions were recruited to form this special task force. During the COVID-19 pandemic, top administrative officers held regular meetings every week and discussed social media strategies including videos, broadcasts, and touching stories to increase public engagement and health literacy. The following may be reasons for the effectiveness of TCVGH’s approach: (1) Top-down leadership in forming the branding team, (2) a high number of Facebook fans who regularly visit the official Facebook page, and (3) various types of posts, such as those including video, to increase engagement. We believe that lessons can be learned from the above experiences.

### Limitations

This study has several limitations. First, we only analyzed data originating from Facebook posts taken from all nationwide medical centers without analyzing any other social media platforms, such as Twitter, Instagram, or LINE. However, Facebook remains the major social media platform with which the Taiwanese people are most familiar. Second, this study was a cross-sectional study, and the content on social media changes every day, so the number of posts or engagement efficacy could have changed by the time the research team accessed the posts. Third, we only enrolled official Facebook fan pages in the analysis. However, we found that some departments within the medical centers had their own Facebook fan page, which usually focused on specific organ systems or diseases. Fourth, classification errors could have occurred because there were 4 types of post category, and it is possible that a given post contained more than one issue. However, classification errors were likely rare because each post categorization was decided by a modified focus group, while a senior division leader served as “gatekeeper” when reviewing the process. Fifth, some data was not made publicly available, such as post frequency, number of views per post, and the changing number of fans at each medical center. That data would improve our understanding of the true effectiveness of various posting strategies. Therefore, further research is warranted to better understand the relationship between dynamic posts and the health outcomes of the people engaged. In addition, some medical centers have more than one official Facebook page; thus, further analysis of multiple Facebook pages within a single hospital may help determine the effectiveness of different Facebook pages set up by different divisions.

### Conclusion

Social media remains an invaluable tool for communication during the COVID-19 pandemic. This nationwide observational study has demonstrated the use of Facebook by academic medical centers in Taiwan and its engagement efficacy. Taiwan continues to play a unique role in the battle against COVID-19, and we would therefore like to share our experience in the use of social media for communication and health promotion, as we believe our knowledge will be helpful to health care organizations worldwide.

## Appendix

Multimedia Appendix 1 COVID-19 Facebook posts of nationwide medical centers and COVID-19 confirmed cases from December 2019 to April 2020 in Taiwan.

Multimedia Appendix 2 The percentage of COVID-19–related Facebook posts by nationwide medical centers from December 2019 to April 2020 in Taiwan.

Multimedia Appendix 3 The odds ratio of COVID-19–related Facebook posts by nationwide medical centers from January to April 2020 in Taiwan.

Multimedia Appendix 4 The relationship of COVID-19 Facebook posts and ownership/region of nationwide medical centers in Taiwan from January to April 2020.

Multimedia Appendix 5 The number of fan likes, comments, and shares on Facebook fan pages, and the percentage of video posts by the central government health agency and 13 nationwide medical centers.

Multimedia Appendix 6 The number of fan likes, comments, and shares on Facebook fan pages, as well as the percentage of video posts published by the central government health agency and 13 nationwide medical centers before and after the COVID-19 outbreak on March 18, 2020.

## Abbreviations

SARS: severe acute respiratory syndrome
TCDC: Taiwan’s Center for Disease Control
JCT: Joint Commission of Taiwan
MOHW: Ministry of Health and Welfare
TCVGH: Taichung Veterans General Hospital
