# Supplementary figures and images for: Use of Facebook by Academic Medical Centers in Taiwan During the COVID-19 Pandemic: Observational Study
Source: J Med Internet Res. 2020 Nov 20;22(11):e21501. doi: 10.2196/21501 (PMC7683023; doi:10.2196/21501)

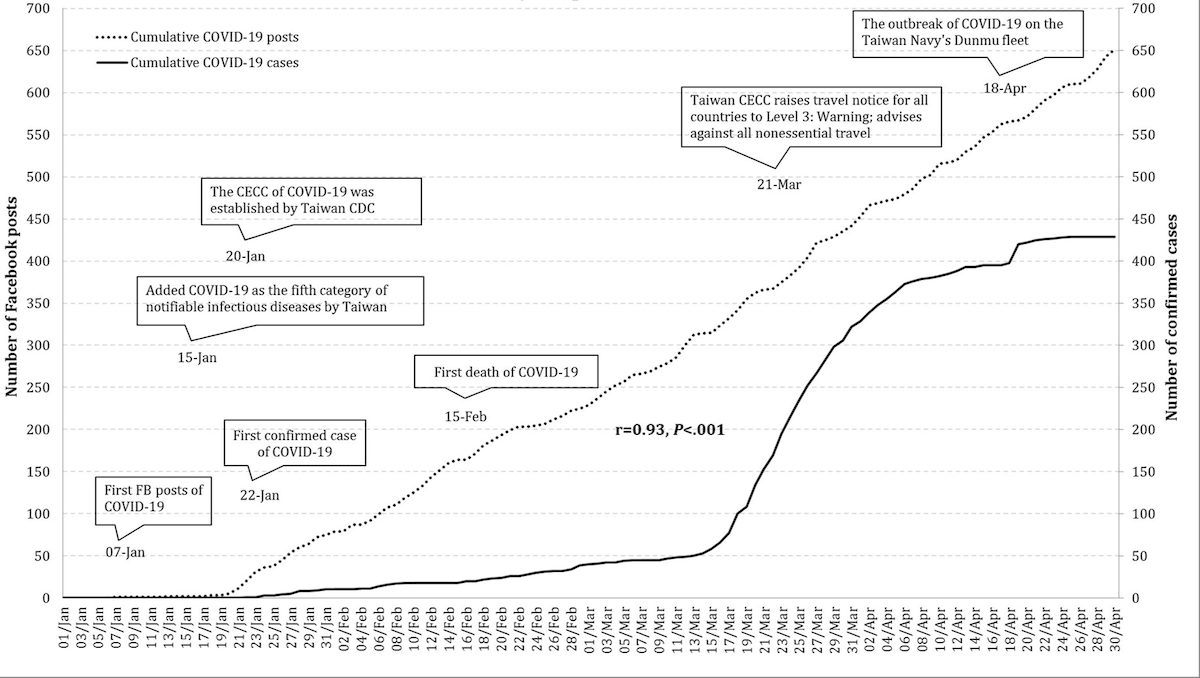

Supplement: Multimedia Appendix 1 [file jmir_v22i11e21501_app1.png]

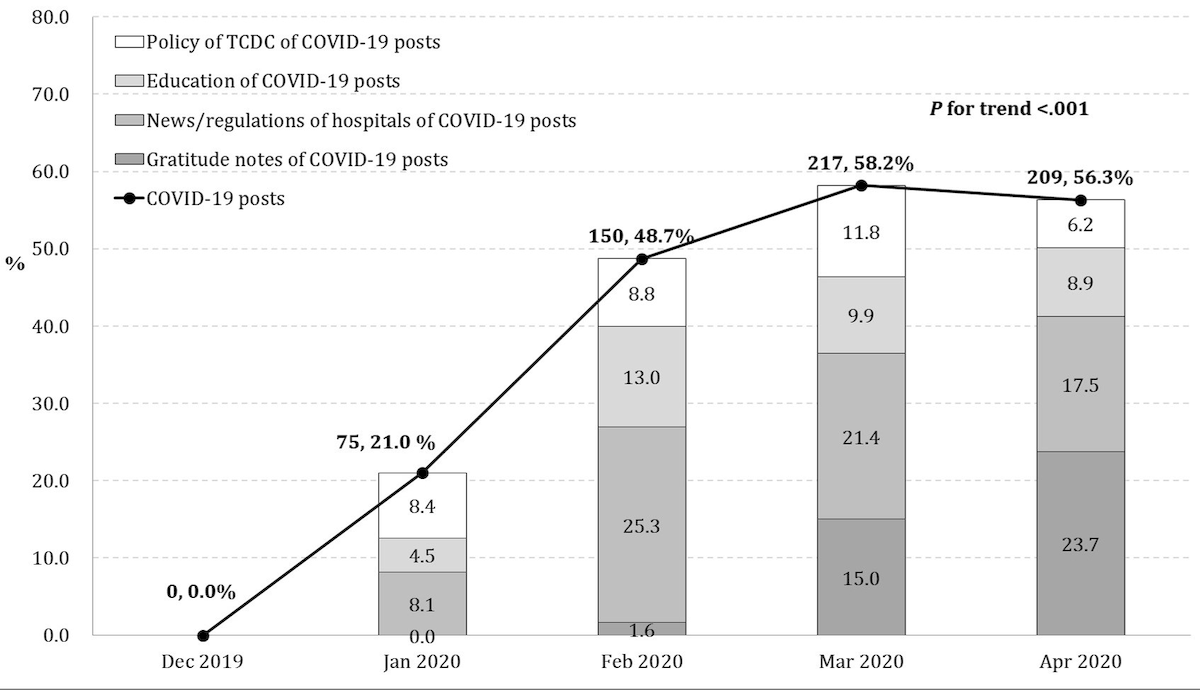

Supplement: Multimedia Appendix 2 [file jmir_v22i11e21501_app2.png]

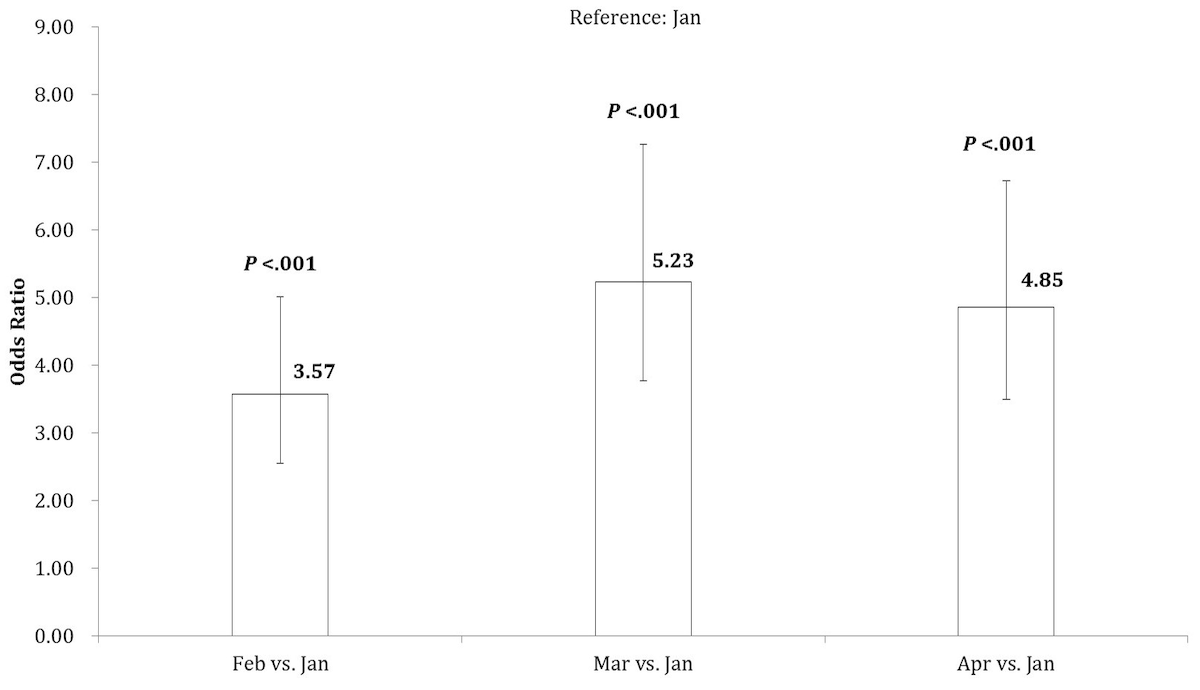

Supplement: Multimedia Appendix 3 [file jmir_v22i11e21501_app3.png]
