# Supplementary material for: Use of Facebook by Academic Medical Centers in Taiwan During the COVID-19 Pandemic: Observational Study
Source: J Med Internet Res. 2020 Nov 20;22(11):e21501. doi: 10.2196/21501 (PMC7683023; doi:10.2196/21501)
Supplement: Multimedia Appendix 4 [file jmir_v22i11e21501_app4.docx]

**Multimedia Appendix 4.** The relationship of COVID-19 Facebook posts and ownership/region of nation-wide medical centers from January to April 2020 in Taiwan.

| Type of post | Item |  | Ownership | | | Region | | | | | | |
| --- | --- | --- | --- | --- | --- | --- | --- | --- | --- | --- | --- | --- |
|  |  |  | Public | Private | *P* value^a^ | North^d^ |  | Central^d^ |  | South^d^ | East^d^ | *P* value^b, c^ |
| Total | Posts | N | 289 | 362 |  | 239 |  | 161 |  | 180 | 71 |  |
|  | Likes | Median(min, max) | 314(28, 19281) | 86(2, 2281) | <.001 | 77(2, 2087) | ^n,cse^ | 197(5, 19281) |  | 213(28, 2281) | 184(34, 1046) | <.001 |
|  | Comment messages | Median(min, max) | 5(0, 2024) | 1(0, 302) | <.001 | 1(0, 2024) | ^n,cs^ | 4(0, 494) | ^c,e^ | 2(0, 195) | 1(0, 173) | <.001 |
|  | Sharing | Median(min, max) | 14(0, 538) | 5(0, 1049) | <.001 | 4(0, 407) |  | 12(0, 469) |  | 10(0, 1049) | 14(0, 123) | <.001 |
|  | Video posts | n(%) | 36(12.5) | 72(19.9) | .011 | 37(15.5) | ^n,cse^ | 39(24.2) | ^c,e^ | 17(9.4) | 15(21.1) | .002 |
|  |  |  |  |  |  |  |  |  |  |  |  |  |
| 1. Policy of TCDC | Posts | N | 55 | 69 |  | 45 |  | 38 |  | 28 | 13 |  |
|  | Likes | Median(min, max) | 236(38, 6679) | 77(2, 356) | <.001 | 158(2, 1187) |  | 52(17, 6679) | ^c,e^ | 112(38, 1004) | 185(108, 356) | .010 |
|  | Comment messages | Median(min, max) | 4(0, 2024) | 0(0, 22) | <.001 | 2(0, 2024) | ^n,c^ | 0(0, 129) |  | 2(0, 10) | 2(0, 9) | .020 |
|  | Sharing | Median(min, max) | 26(0, 469) | 8(0, 191) | <.001 | 22(0, 407) |  | 5(1, 469) | ^c,e^ | 18(0, 199) | 34(7, 123) | .007 |
|  | Video | n(%) | 0(0.0) | 4(5.8) | .070 | 1(2.2) |  | 3(7.9) |  | 0(0.0) | 0(0.0) | .244 |
|  |  |  |  |  |  |  |  |  |  |  |  |  |
| 2. Gratitude notes | Posts | N | 72 | 77 |  | 73 |  | 26 |  | 37 | 13 |  |
|  | Likes | Median(min, max) | 359(39, 2237) | 138(12, 419) | <.001 | 207(12, 2087) |  | 225(75, 2237) |  | 229(39, 1254) | 183(77, 419) | .024 |
|  | Comment messages | Median(min, max) | 5(0, 199) | 1(0, 39) | <.001 | 2(0, 199) | ^n,c^ | 8(0, 89) | ^c,e^ | 4(0, 45) | 1(0, 7) | .001 |
|  | Sharing | Median(min, max) | 5(0, 132) | 3(0, 55) | .004 | 3(0, 61) | ^n,cs^ | 8(0, 49) | ^c,e^ | 5(0, 132) | 4(0, 16) | .001 |
|  | Video | n(%) | 3(4.2) | 15(19.5) | 0.004 | 9(12.3) |  | 5(19.2) |  | 4(10.8) | 0(0.0) | .377 |
|  |  |  |  |  |  |  |  |  |  |  |  |  |
| 3. News/regulations of hospitals | Posts | N | 107 | 145 |  | 88 |  | 50 |  | 85 | 29 |  |
|  | Likes | Median(min, max) | 350(28, 19281) | 78(3, 2281) | <.001 | 45(3, 1087) | ^n,cse^ | 229(5, 19281) |  | 281(28, 2281) | 202(34, 1046) | <.001 |
|  | Comment messages | Median(min, max) | 6(0, 494) | 1(0, 195) | <.001 | 0(0, 52) | ^n,cs^ | 8(0, 494) |  | 4(0, 195) | 1(0, 173) | <.001 |
|  | Sharing | Median(min, max) | 21(0, 538) | 6(0, 1049) | <.001 | 5(0, 152) | ^n,cse^ | 17(0, 259) |  | 15(0, 1049) | 12(0, 104) | <.001 |
|  | Video | n(%) | 13(12.1) | 34(23.4) | .021 | 19(21.6) |  | 12(24.0) |  | 8(9.4) | 8(27.6) | .016 |
|  |  |  |  |  |  |  |  |  |  |  |  |  |
| 4. Education | Posts | N | 55 | 71 |  | 33 |  | 47 |  | 30 | 16 |  |
|  | Likes | Median(min, max) | 282(48, 1746) | 63(3, 544) | <.001 | 35(3, 138) | ^n,cse^ | 321(37, 1601) |  | 110(48, 1746) | 104(39, 318) | <.001 |
|  | Comment messages | Median(min, max) | 4(0, 103) | 0(0, 302) | .001 | 0(0, 51) |  | 7(0, 302) | ^c,e^ | 1(0, 97) | 1(0, 17) | <.001 |
|  | Sharing | Median(min, max) | 13(0, 369) | 6(0, 77) | .001 | 2(0, 12) | ^n,cse^ | 17(0, 172) |  | 8(1, 369) | 18(1, 31) | <.001 |
|  | Video | n(%) | 20(36.4) | 19(26.8) | .248 | 8(24.2) |  | 19(40.4) |  | 5(16.7) | 7(43.8) | .080 |

^a^Mann-Whitney U test.

^b^Continuous variable using Kruskal-Wallis test and Dunn's post hoc test for differences with a significant between the region.

^c^Categorical variable using hi-square test and and Bonferroni correction post hoc tests for differences with a significant between the region.

^d^With Dunn's post hoc test for pair‐wise region comparisons, the initials "n, cse" indicates that the northern hospital is different from the central, southern, eastern hospital and so on.
