# Supplementary material for: Use of Facebook by Academic Medical Centers in Taiwan During the COVID-19 Pandemic: Observational Study
Source: J Med Internet Res. 2020 Nov 20;22(11):e21501. doi: 10.2196/21501 (PMC7683023; doi:10.2196/21501)
Supplement: Multimedia Appendix 6 [file jmir_v22i11e21501_app6.docx]

Multimedia Appendix 6. Before and after the COVID-19 outbreak on March 18, the number of fans likes, comment messages, sharing, and the percentage of video posts on Facebook of the central government health agency and nationwide 13 medical centers.

| Region/  Ownership | Institution name | Type of post | Before the COVID-19 outbreak on March 18th | | | | | |  | After COVID-19 outbreak on March 18th | | | | | |  | *P* value | | | |
| --- | --- | --- | --- | --- | --- | --- | --- | --- | --- | --- | --- | --- | --- | --- | --- | --- | --- | --- | --- | --- |
|  |  |  | Posts | Likes | Comment messages | Sharing | Video posts | |  | Posts | Likes | Comment messages | Sharing | Video posts | |  | Likes^a^ | Comment messages^a^ | Sharing^a^ | Video posts^b^ |
|  |  |  |  |  |  |  |  |  |  |  |  |  |  |  |  |  |  |  |  |  |
|  |  |  | n | Median | Median | Median | n | (%) |  | n | Median | Median | Median | n | (%) |  |  |  |  |  |
| Central government health agency | Ministry of Health and Welfare (MOHW) | Total | 299 | 4,561 | 142 | 343 | 2 | (0.7) |  | 287 | 8,409 | 272 | 387 | 5 | (1.7) |  | <.001 | <.001 | 0.085 | 0.232 |
|  |  | 1.Policy of TCDC | 286 | 4,438 | 140 | 346 | 2 | (0.7) |  | 228 | 8,517 | 304 | 389 | 5 | (2.2) |  | <.001 | <.001 | 0.046 | 0.147 |
|  |  | 2.Gratitude notes | 5 | 1,5033 | 410 | 394 | 0 | (-) |  | 5 | 19,008 | 110 | 1,359 | 0 | (-) |  | 1.000 | 0.676 | 0.531 |  |
|  |  | 3.News/regulations of hospitals | 0 | - | - | - | - | (-) |  | 0 | - | - | - | - | (-) |  |  |  |  |  |
|  |  | 4.Education | 8 | 6,396 | 108 | 239 | 0 | (-) |  | 54 | 7,770 | 203 | 308 | 0 | (-) |  | 0.176 | 0.045 | 0.713 |  |
|  | Taiwan Centers of Disease Control (TCDC) | Total | 332 | 2,169 | 138 | 217 | 27 | (8.1) |  | 164 | 3,499 | 192 | 140 | 32 | (19.5) |  | <.001 | 0.038 | 0.013 | <.001 |
|  |  | 1.Policy of TCDC | 318 | 2,132 | 137 | 200 | 27 | (8.5) |  | 150 | 3,364 | 199 | 131 | 31 | (20.7) |  | <.001 | 0.014 | 0.002 | <.001 |
|  |  | 2.Gratitude notes | 3 | 1,1703 | 949 | 84 | 0 | (-) |  | 1 | 2,805 | 162 | 138 | 0 | (-) |  | 0.346 | 0.346 | 1.000 |  |
|  |  | 3.News/regulations of hospitals | 0 | - | - | - | - | (-) |  | 0 | - | - | - | - | (-) |  |  |  |  |  |
|  |  | 4.Education | 11 | 2,538 | 138 | 402 | 0 | (-) |  | 13 | 4,931 | 112 | 530 | 1 | (7.7) |  | 0.049 | 0.977 | 0.486 | 1.000 |
| Nationwide 13 medical centers | | Total | 332 | 332 | 136 | 1 | 10 | (14.8) |  | 319 | 166 | 2 | 7 | 59 | (18.5) |  | 0.026 | 0.042 | <.001 | 0.200 |
|  |  | 1.Policy of TCDC | 84 | 84 | 112 | 1 | 14 | (3.6) |  | 40 | 150 | 2 | 14 | 1 | (2.5) |  | 0.220 | 0.440 | 0.642 | 0.752 |
|  |  | 2.Gratitude notes | 19 | 19 | 218 | 4 | 5 | (21.1) |  | 130 | 219 | 2 | 4 | 14 | (10.8) |  | 0.829 | 0.184 | 0.151 | 0.199 |
|  |  | 3.News/regulations of hospitals | 153 | 153 | 192 | 2 | 13 | (15.0) |  | 99 | 126 | 2 | 10 | 24 | (24.2) |  | 0.407 | 0.907 | 0.175 | 0.067 |
|  |  | 4.Education | 76 | 76 | 88 | 0 | 8 | (25.0) |  | 50 | 131 | 4 | 8 | 20 | (40.0) |  | 0.040 | 0.005 | 0.855 | 0.075 |
| Northern public | National Taiwan University Hospital | Total | 12 | 199 | 3 | 33 | 0 | (-) |  | 13 | 142 | 13 | 28 | 0 | (-) |  | 0.356 | 0.146 | 0.663 |  |
|  |  | 1.Policy of TCDC | 7 | 364 | 5 | 71 | 0 | (-) |  | 5 | 211 | 22 | 41 | 0 | (-) |  | 0.516 | 0.143 | 0.626 |  |
|  |  | 2.Gratitude notes | 0 | - | - | - | - | (-) |  | 1 | 393 | 199 | 11 | 0 | (-) |  |  |  |  |  |
|  |  | 3.News/regulations of hospitals | 4 | 116 | 3 | 7 | 0 | (-) |  | 6 | 122 | 3 | 21 | 0 | (-) |  | 1.000 | 0.912 | 0.285 |  |
|  |  | 4.Education | 1 | 71 | 0 | 4 | 0 | (-) |  | 1 | 97 | 51 | 3 | 0 | (-) |  |  |  |  |  |
|  | Tri-Service General Hospital | Total | 12 | 152 | 1 | 7 | 0 | (-) |  | 53 | 284 | 3 | 4 | 0 | (-) |  | 0.019 | 0.194 | 0.312 |  |
|  |  | 1.Policy of TCDC | 5 | 204 | 2 | 19 | 0 | (-) |  | 7 | 179 | 1 | 5 | 0 | (-) |  | 1.000 | 0.673 | 0.253 |  |
|  |  | 2.Gratitude notes | 0 | - | - | - | - | (-) |  | 38 | 300 | 4 | 4 | 0 | (-) |  |  |  |  |  |
|  |  | 3.News/regulations of hospitals | 4 | 190 | 2 | 10 | 0 | (-) |  | 7 | 320 | 2 | 13 | 0 | (-) |  | 0.449 | 0.633 | 0.445 |  |
|  |  | 4.Education | 3 | 131 | 0 | 3 | 0 | (-) |  | 1 | 127 | 0 | 1 | 0 | (-) |  | 1.000 |  | 0.371 |  |
|  | Taipei Veterans General Hospital | Total | 1 | 166 | 16 | 34 | 0 | (-) |  | 3 | 151 | 3 | 16 | 0 | (-) |  | 1.000 | 0.371 | 0.637 |  |
|  |  | 1.Policy of TCDC | 1 | 166 | 16 | 34 | 0 | (-) |  | 2 | 184 | 3 | 21 | 0 | (-) |  | 1.000 | 0.540 | 1.000 |  |
|  |  | 2.Gratitude notes | 0 | - | - | - | - | (-) |  | 0 | - | - | - | - | (-) |  |  |  |  |  |
|  |  | 3.News/regulations of hospitals | 0 | - | - | - | - | (-) |  | 1 | 113 | 8 | 16 | 0 | (-) |  |  |  |  |  |
|  |  | 4.Education | 0 | - | - | - | - | (-) |  | 0 | - | - | - | - | (-) |  |  |  |  |  |
| Northern private | MacKay Memorial Hospital | Total | 2 | 244 | 11 | 67 | 0 | (-) |  | 3 | 184 | 5 | 78 | 0 | (-) |  | 1.000 | 0.767 | 0.773 |  |
|  |  | 1.Policy of TCDC | 1 | 168 | 11 | 29 | 0 | (-) |  | 2 | 222 | 14 | 135 | 0 | (-) |  | 0.540 | 1.000 | 0.540 |  |
|  |  | 2.Gratitude notes | 0 | - | - | - | - | (-) |  | 0 | - | - | - | - | (-) |  |  |  |  |  |
|  |  | 3.News/regulations of hospitals | 1 | 319 | 11 | 105 | 0 | (-) |  | 1 | 184 | 4 | 39 | 0 | (-) |  |  |  |  |  |
|  |  | 4.Education | 0 | - | - | - | - | (-) |  |  | - | - | - | - | (-) |  |  |  |  |  |
|  | Shin Kong Wu Ho-Su Memorial Hospital | Total | 16 | 6 | 0 | 0 | 8 | (50.0) |  | 41 | 27 | 0 | 0 | 7 | (17.1) |  | <.001 | 0.017 | 0.039 | 0.019 |
|  |  | 1.Policy of TCDC | 1 | 9 | 0 | 0 | 1 | (100.0) |  | 1 | 22 | 0 | 0 | 0 | (-) |  |  |  |  | 1.000 |
|  |  | 2.Gratitude notes | 2 | 31 | 0 | 2 | 0 | (-) |  | 21 | 33 | 0 | 0 | 5 | (23.8) |  | 0.743 | 0.435 | 0.214 | 1.000 |
|  |  | 3.News/regulations of hospitals | 12 | 5 | 0 | 0 | 7 | (58.3) |  | 14 | 9 | 0 | 0 | 1 | (7.1) |  | 0.003 | 0.054 | 0.027 | 0.009 |
|  |  | 4.Education | 1 | 6 | 0 | 0 | 0 | (-) |  | 5 | 22 | 0 | 1 | 1 | (20.0) |  | 0.242 |  | 0.534 | 1.000 |
|  | Taipei Municipal Wanfang Hospital | Total | 12 | 33 | 0 | 4 | 2 | (16.7) |  | 3 | 347 | 11 | 34 | 0 | (-) |  | 0.017 | 0.056 | 0.011 | 1.000 |
|  |  | 1.Policy of TCDC | 4 | 33 | 4 | 5 | 0 | (-) |  | 2 | 227 | 7 | 72 | 0 | (-) |  | 0.100 | 0.481 | 0.105 |  |
|  |  | 2.Gratitude notes | 1 | 248 | 12 | 12 | 0 | (-) |  | 1 | 347 | 26 | 25 | 0 | (-) |  |  |  |  |  |
|  |  | 3.News/regulations of hospitals | 3 | 29 | 2 | 3 | 0 | (-) |  | 0 | - | - | - | - | (-) |  |  |  |  |  |
|  |  | 4.Education | 4 | 31 | 0 | 4 | 2 | (50.0) |  | 0 | - | - | - | - | (-) |  |  |  |  |  |
|  | Far Eastern Memorial Hospital | Total | 41 | 30 | 0 | 5 | 7 | (17.1) |  | 27 | 89 | 2 | 6 | 13 | (48.1) |  | <.001 | 0.000 | 0.030 | 0.006 |
|  |  | 1.Policy of TCDC | 5 | 15 | 0 | 1 | 0 | (-) |  | 2 | 73 | 1 | 12 | 0 | (0.0) |  | 0.333 | 0.624 | 0.329 |  |
|  |  | 2.Gratitude notes | 0 | - | - | - | - | (-) |  | 9 | 128 | 4 | 5 | 4 | (44.4) |  |  |  |  |  |
|  |  | 3.News/regulations of hospitals | 23 | 39 | 0 | 5 | 4 | (17.4) |  | 12 | 72 | 2 | 13 | 7 | (58.3) |  | 0.004 | 0.066 | 0.109 | 0.022 |
|  |  | 4.Education | 13 | 27 | 0 | 2 | 3 | (23.1) |  | 4 | 88 | 0 | 4 | 2 | (50.0) |  | 0.005 | 0.609 | 0.529 | 0.538 |
| Central public | Taichung Veterans General Hospital | Total | 37 | 674 | 22 | 32 | 12 | (32.4) |  | 29 | 589 | 14 | 16 | 15 | (51.7) |  | 0.311 | 0.091 | 0.004 | 0.114 |
|  |  | 1.Policy of TCDC | 8 | 609 | 13 | 34 | 0 | (-) |  | 0 | - | - | - | - | (-) |  |  |  |  |  |
|  |  | 2.Gratitude notes | 2 | 1494 | 61 | 27 | 0 | (-) |  | 7 | 781 | 18 | 13 | 0 | (-) |  | 0.464 | 0.056 | 1.000 |  |
|  |  | 3.News/regulations of hospitals | 12 | 954 | 31 | 69 | 4 | (33.3) |  | 10 | 621 | 16 | 19 | 6 | (60.0) |  | 0.113 | 0.106 | 0.120 | 0.391 |
|  |  | 4.Education | 15 | 518 | 13 | 29 | 8 | (53.3) |  | 12 | 507 | 13 | 21 | 9 | (75.0) |  | 0.961 | 0.449 | 0.435 | 0.424 |
| Central private | Chung Shan Medical University Hospital | Total | 53 | 74 | 0 | 7 | 5 | (9.4) |  | 42 | 121 | 0 | 7 | 7 | (16.7) |  | 0.014 | 0.280 | 0.206 | 0.292 |
|  |  | 1.Policy of TCDC | 21 | 40 | 0 | 4 | 2 | (9.5) |  | 9 | 78 | 0 | 5 | 1 | (11.1) |  | 0.031 | 0.437 | 0.479 | 1.000 |
|  |  | 2.Gratitude notes | 5 | 134 | 4 | 5 | 3 | (60.0) |  | 12 | 170 | 3 | 4 | 2 | (16.7) |  | 0.225 | 0.592 | 0.313 | 0.117 |
|  |  | 3.News/regulations of hospitals | 16 | 160 | 1 | 16 | 0 | (-) |  | 12 | 80 | 0 | 6 | 2 | (16.7) |  | 0.041 | 0.114 | 0.318 | 0.175 |
|  |  | 4.Education | 11 | 79 | 0 | 13 | 0 | (-) |  | 9 | 169 | 5 | 8 | 2 | (22.2) |  | 0.081 | 0.066 | 0.127 | 0.190 |
| Southern public | National Cheng Kung University Hospital | Total | 44 | 400 | 7 | 30 | 4 | (9.1) |  | 19 | 390 | 4 | 21 | 0 | (-) |  | 0.708 | 0.263 | 0.345 | 0.306 |
|  |  | 1.Policy of TCDC | 3 | 370 | 5 | 70 | 0 | (-) |  | 1 | 390 | 2 | 89 | 0 | (-) |  | 1.000 | 1.000 | 1.000 |  |
|  |  | 2.Gratitude notes | 2 | 833 | 12 | 69 | 0 | (-) |  | 3 | 508 | 5 | 9 | 0 | (-) |  | 0.773 | 0.773 | 0.773 |  |
|  |  | 3.News/regulations of hospitals | 34 | 468 | 8 | 30 | 3 | (8.8) |  | 11 | 369 | 4 | 30 | 0 | (-) |  | 0.421 | 0.341 | 0.561 | 0.565 |
|  |  | 4.Education | 5 | 282 | 1 | 27 | 1 | (20.0) |  | 4 | 387 | 3 | 24 | 0 | (-) |  | 0.903 | 0.898 | 1.000 | 1.000 |
|  | Kaohsiung Veterans General Hospital | Total | 42 | 134 | 2 | 10 | 1 | (2.4) |  | 24 | 193 | 3 | 6 | 4 | (16.7) |  | 0.018 | 0.042 | 0.106 | 0.055 |
|  |  | 1.Policy of TCDC | 12 | 86 | 1 | 13 | 0 | (-) |  | 4 | 253 | 3 | 20 | 0 | (-) |  | 0.302 | 0.417 | 0.395 |  |
|  |  | 2.Gratitude notes | 7 | 218 | 4 | 3 | 1 | (14.3) |  | 12 | 396 | 7 | 6 | 2 | (16.7) |  | 0.118 | 0.497 | 0.418 | 1.000 |
|  |  | 3.News/regulations of hospitals | 15 | 190 | 2 | 17 | 0 | (-) |  | 3 | 145 | 2 | 2 | 0 | (0.0) |  | 0.477 | 1.000 | 0.033 |  |
|  |  | 4.Education | 8 | 67 | 0 | 7 | 0 | (-) |  | 5 | 105 | 1 | 5 | 2 | (40.0) |  | 0.107 | 0.005 | 0.419 | 0.128 |
| Southern private | Kaohsiung Medical University Chung-Ho Memorial Hospital | Total | 23 | 89 | 0 | 8 | 1 | (4.3) |  | 28 | 163 | 3 | 6 | 7 | (25.0) |  | <.001 | 0.004 | 0.352 | 0.059 |
|  |  | 1.Policy of TCDC | 6 | 94 | 0 | 11 | 0 | (-) |  | 2 | 151 | 3 | 10 | 0 | (-) |  | 0.868 | 0.214 | 1.000 |  |
|  |  | 2.Gratitude notes | 0 | - | - | - | - | () |  | 13 | 180 | 2 | 4 | 1 | (7.7) |  |  |  |  |  |
|  |  | 3.News/regulations of hospitals | 11 | 89 | 0 | 8 | 0 | (-) |  | 11 | 156 | 4 | 4 | 5 | (45.5) |  | 0.010 | 0.020 | 0.643 | 0.035 |
|  |  | 4.Education | 6 | 91 | 1 | 7 | 1 | (16.7) |  | 2 | 164 | 6 | 8 | 1 | (50.0) |  | 0.067 | 0.129 | 0.736 | 0.464 |
| Eastern private | Hualien Tzu Chi Hospital | Total | 37 | 206 | 2 | 17 | 9 | (24.3) |  | 34 | 150 | 1 | 11 | 6 | (17.6) |  | 0.022 | 0.251 | 0.073 | 0.491 |
|  |  | 1.Policy of TCDC | 10 | 211 | 3 | 32 | 0 | (-) |  | 3 | 171 | 1 | 45 | 0 | (-) |  | 0.076 | 0.606 | 0.933 |  |
|  |  | 2.Gratitude notes | 0 | - | - | - | - | (-) |  | 13 | 183 | 1 | 4 | 0 | (-) |  |  |  |  |  |
|  |  | 3.News/regulations of hospitals | 18 | 216 | 2 | 10 | 5 | (27.8) |  | 11 | 160 | 1 | 14 | 3 | (27.3) |  | 0.144 | 0.404 | 0.458 | 1.000 |
|  |  | 4.Education | 9 | 142 | 1 | 19 | 4 | (44.4) |  | 7 | 88 | 1 | 15 | 3 | (42.9) |  | 0.169 | 0.588 | 0.314 | 1.000 |

Note: If the number of Facebook posts is zero, the table cell is represented by the symbol dash. Blank cells indicate that unable to perform statistical testing.

^a^ Mann-Whitney U test

^b^ Chi-square test, but if one of the expected counts in the 2x2 contingency table is less than 5, using the Fisher's exact test.
